# Building a European exposure science strategy

**DOI:** 10.1038/s41370-019-0193-7

**Published:** 2019-12-02

**Authors:** Peter Fantke, Natalie von Goetz, Urs Schlüter, Jos Bessems, Alison Connolly, Tatsiana Dudzina, Andreas Ahrens, Jim Bridges, Marie A. Coggins, André Conrad, Otto Hänninen, Gerhard Heinemeyer, Stylianos Kephalopoulos, Michael McLachlan, Tim Meijster, Veronique Poulsen, Dag Rother, Theo Vermeire, Susana Viegas, Jelle Vlaanderen, Maryam Zare Jeddi, Yuri Bruinen de Bruin

**Affiliations:** 1 Quantitative Sustainability Assessment, Department of Technology, Management and Economics, Technical University of Denmark, Produktionstorvet 424, 2800 Kgs, Lyngby, Denmark; 2 Federal Office of Public Health, Köniz, Switzerland; 3 Federal Institute for Occupational Safety and Health, Dortmund, Germany; 4 Flemish Institute for Technological Research, Mol, Belgium; 5 School of Physics and the Ryan Institute, National University of Ireland, Galway, Ireland; 6 ExxonMobil, Brussels, Belgium; 7 European Chemicals Agency, Helsinki, Finland; 8 Research for Sustainability, University of Surrey, Guildford, UK; 9 German Environment Agency, Dessau-Roßlau, Germany; 10 THL Public Health Solutions, Kuopio, Finland; 11 German Federal Institute for Risk Assessment, Berlin, Germany; 12 European Commission, Joint Research Centre, Directorate F—Health, Consumers and Reference Materials, Ispra, Italy; 13 Stockholm University, Stockholm, Sweden; 14 Shell/ECETOC, Utrecht, Netherlands; 15 L’Oréal, Clichy, France; 16 National Institute for Public Health and the Environment, Utrecht, Netherlands; 17 H&TRC Health & Technology Research Center, ESTeSL Escola Superior de Tecnologia da Saúde, Instituto Politécnico de Lisboa, Lisbon, Portugal; 18 CISP Centro de Investigação em Saúde Pública, Escola Nacional de Saúde Pública, Universidade NOVA de Lisboa, Lisbon, Portugal; 19 Institutes for Risk Assessment Sciences, Utrecht University, Utrecht, Netherlands; 20 Department of Cardio-Thoraco-Vascular Sciences and Public Health, University of Padua, Padua, Italy; 21 European Commission, Joint Research Centre, Directorate E—Space, Security and Migration, Ispra, Italy

**Keywords:** ISES Europe, Human exposure, Environmental exposure, Exposure assessment, Risk assessment, International Society of Exposure Science

## Abstract

Exposure information is a critical element in various regulatory and non-regulatory frameworks in Europe and elsewhere. Exposure science supports to ensure safe environments, reduce human health risks, and foster a sustainable future. However, increasing diversity in regulations and the lack of a professional identity as exposure scientists currently hamper developing the field and uptake into European policy. In response, we discuss trends, and identify three key needs for advancing and harmonizing exposure science and its application in Europe. We provide overarching building blocks and define six long-term activities to address the identified key needs, and to iteratively improve guidelines, tools, data, and education. More specifically, we propose creating European networks to maximize synergies with adjacent fields and identify funding opportunities, building common exposure assessment approaches across regulations, providing tiered education and training programmes, developing an aligned and integrated exposure assessment framework, offering best practices guidance, and launching an exposure information exchange platform. Dedicated working groups will further specify these activities in a consistent action plan. Together, these elements form the foundation for establishing goals and an action roadmap for successfully developing and implementing a ‘European Exposure Science Strategy’ 2020–2030, which is aligned with advances in science and technology.

## Exposure science as an important discipline

Exposure science is gaining more and more attention worldwide as an important discipline focussing on stressors occurring in or released to the natural and man-made environment, and their potential hazards for humans and ecosystems. Various foundational National Research Council reports recognise exposure science as an essential and integral part of the risk management process [1–3]. Compared with toxicology, however, exposure science is still ill developed as an independent discipline [2, 4]. Yet, it has become an indispensable element in various science and policy frameworks, from policy analysis to sustainability assessment [5–13], and has been proposed as an important component in chemical substitution [14, 15]. Although many regulations and studies focus on exposure to chemicals, assessing and managing exposure to other stressors is increasingly acknowledged. This includes biological (e.g. bacterial, fungal, viral, and parasitic agents) [16] and physical stressors (e.g. climatic factors, noise, and vibration) [17, 18]. This broad scope of exposure science is also reflected in its various definitions (e.g. [2, 19]), explicitly going beyond chemical stressors and humans as target receptors (see Textbox 1).

At its core, exposure science contributes to an improved understanding of stressor-health-environment relationships. It furthermore contributes to achieving several global exposure reduction and environmental protection targets [8, 21, 22]. With that, exposure science constitutes a growing science field with a broad applicability range. Nevertheless, substantial efforts remain to develop its full potential to become a viable part in enabling decision-making for safe and secure, sustainable, and healthy societies and environments.

Textbox 1. Working definition of exposure science [20], adapted and expanded from definitions discussed elsewhere [2, 19]. Exposure science studies the contact between stressors and receptors, and the associated sources, pathways and processes potentially leading to impacts on human health and the natural and built environment.

Stressors primarily refer to chemical, biological, and physical agents, and receptors range from molecules, cells, and organs to humans and other organisms.

## The European context

**Fig. 1:**
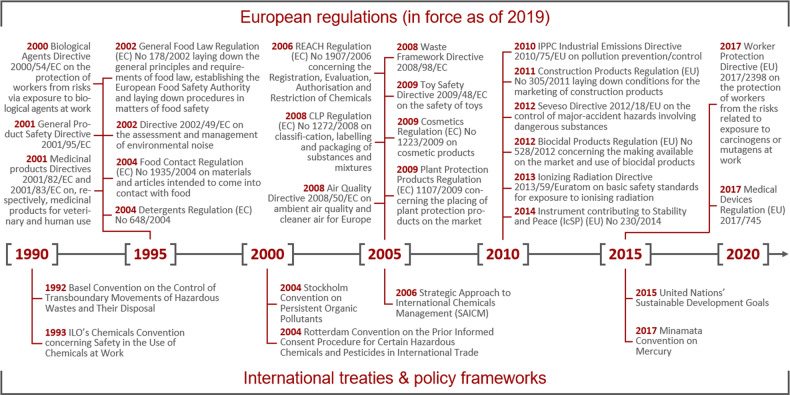
List of major European regulations, and international treaties and policy frameworks currently in force, where exposure information in relation to different stressors is an essential element. Note that only those regulations are listed that are still in force, while exposure information might have been already introduced in preceding regulations (e.g. Council Directive 91/414/EEC for Plant Protection Products)

In Europe, exposure science is strongly anchored in different regulatory frameworks, each creating specific demands and requirements for exposure information. The scattered policy landscape represents a substantial challenge for practitioners and decision-makers for developing and harmonising exposure data, methods, and tools. The current European regulation concerning the Registration, Evaluation, Authorisation, and Restriction of Chemicals (REACH) [23] created a fundamental mandate to deliver adequate exposure information to foster the safe use of chemicals in the European Economic Area. REACH thereby applies to all substances manufactured or imported into the EU at a scale of at least 1 tonne per year, and covers all uses (as such, in mixtures or in articles) unless explicitly regulated elsewhere. This was driven by the need for high quality and reliable information on marketed chemicals—including data on use patterns of substances and products, and related human and ecological exposure [24]. Reliable exposure information is also required by many other European regulations ranging from product safety to waste management (Fig. 1), and even in stabilisation and peace-building instruments [25]. While most regulations focus on chemical exposure [26], exposure science faces increasing demands for considering areas beyond the scope of chemicals and classical human and environmental risk assessment. This regulatory diversity constitutes a challenge for industry, which faces additional exposure-related reporting requirements in support of more holistic risk management processes despite the aim of regulations like REACH to streamline the management of industry’s reporting burden.

Beyond the regulatory context, exposure science is essential for several recently proposed European strategies, from the development of nature-based solutions to resources efficiency based on life cycle approaches [27, 28]. A special challenge for the latter is to appropriately consider all life cycle stages of a given product when, for example, characterising exposure to air pollutants [29, 30] or to chemicals in consumer products [31–33]. Other European strategies include moving towards a bio-based and circular economy, promoting sustainable chemistry and a non-toxic environment by 2050, and creating a protecting Europe [34–38]. All these strategies represent additional challenges for providing adequate exposure information.

Exposure information is finally also relevant in international treaties focussing on hazardous chemicals and waste, in the global policy framework ‘Strategic Approach to International Chemicals Management’, and in several targets set in the United Nations’ Sustainable Development Goals (Fig. 1).

Overall, regulatory changes over the past two decades along with the Commission’s ambitions to move towards a safe, secure, and sustainable Europe [39, 40] has increased the demand for high-quality exposure information. This has led to distinct approaches, for example, to manage insufficient or missing exposure information in different regulatory and non-regulatory decision contexts, such as introducing ‘default’ values and generic assumptions. However, such values are often not well underpinned by evidence, which can lead to over- or underestimated exposure (and related risks or impacts), which in turn compromises and questions the reliability of regulatory and other decisions [41].

The needs to close gaps and align regulatory and non-regulatory requirements on exposure information have put a unique demand on the European exposure science community. This currently hampers the uptake of advances in the field via a systematic and harmonised approach, streamlined into common exposure assessment and management practices in support of implementing European policies and strategies [42]. An overarching alignment effect is hence required for addressing the current and future needs for exposure science in Europe, and for developing a roadmap for generating, harmonizing, and effectively applying exposure information in European frameworks. Such efforts are being developed in specific subdomains, for example in the EU-funded HBM4EU project, aiming to ‘advance human biomonitoring in Europe to provide evidence for chemical policy making’ [43]. Yet, an overarching strategy is needed, which stimulates synergies across subdomains, respects Europe’s ambitions and regulatory requirements, is aligned with global societal goals for human and environmental health, while continuously integrating knowledge from major scientific trends.

## Considering major scientific trends

Generating exposure information is influenced by several major trends in, for example, data analytics, machine learning, and citizen science. Along such trends, we illustrate important achievements for exposure science, which seeks to evolve as an independent and strong scientific discipline. One trend is the increased ambition for a holistic understanding of the complex interactions between stressors, products, technologies, humans, and the environment. Related emerging topics are the ‘exposome’ concept [44, 45] or ‘safe-by-design’ initiatives [46]. Underlying exposure data generation increasingly builds on high-throughput and spatial methods [47–51], and harmonized biomonitoring efforts [43, 52], which will influence the availability, quality and complexity of exposure information. Various important achievements including exposomics, intake fraction modelling, and personal exposure sampling are further detailed elsewhere (e.g. [2]).

Advancing exposure science in Europe should systematically build on such achievements, considering the following four principles. First, exposure science offers knowledge, which goes beyond safety and also addresses security and sustainability aspects [2]. Second, global trends to move away from animal testing increase the dependency on alternative exposure estimation methods, enabling innovative and exposure-based ‘intelligent or integrated testing strategies’ (ITS) [53]. Third, there is a growing emphasis on addressing the complex human-environment interactions within a globalised economy perspective, considering circular material flows and supply chains [8, 54]. Fourth, the need for assessing complex exposures [2, 19] and benchmarking exposure estimates against health capacity limits [55, 56] are increasingly recognised, while respective data and methods are currently missing. Following these principles needs to build on identifying relevant funding opportunities from industry, such as the Long-Range Research Initiative (LRI) of the European Chemical Industry Council (CEFIC), and from major European research programs, such as Horizon Europe [46]. Such a development for exposure science in Europe requires an overarching *European Exposure Science Strategy* to ensure a consolidated and successful effort. The main elements for building such a strategy are outlined in the following.

## Elements for building a strategy

**Fig. 2:**
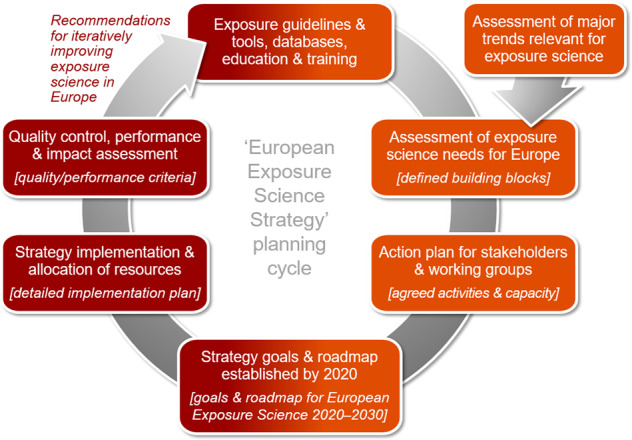
Process cycle for building a European Exposure Science Strategy by 2020 with definition of goals and roadmap, and implementing the strategy in Europe during 2020–2030 as key points to harmonise and improve exposure guidelines and tools, databases, and education and training activities in Europe

Building and implementing an overarching strategy for advancing exposure science in Europe needs to start from establishing an active professional ‘community of practice’. This community would have the mandate to develop missing guidance in relation to exposure knowledge and in relation to identifying the degree of representativeness, applicability and quality of exposure estimates in various decision contexts. As platform for such a professional exposure science community in Europe, the *Europe Regional Chapter of the International Society of Exposure Science* (ISES Europe, http://ises-europe.org) was founded in 2017 as a Regional Chapter of ISES to better meet the needs and practices with regard to exposure research and policies in Europe [57]. In line with the recent European and international developments (see also Fig. 1), initial focus should be on specific advancements and harmonization efforts across different regulations and sectors. To align such efforts, it is critical to develop consistent goals and a clear roadmap for a common European Exposure Science Strategy and to broadly support its implementation. Long-term vision of such a strategy is to foster the consistent and continuous uptake of exposure science in Europe across regulatory and non-regulatory frameworks and by the various stakeholders. Building this strategy has to meet two conditions. First, the starting point must be a set of identified needs for exposure science in Europe and aligned activities to address these needs. Second, defining and implementing a roadmap of activities and their specific tasks requires an iterative process to address possible changes in key needs, major trends and funding opportunities. The iterative process for building and implementing this strategy is presented in Fig. 2. The first elements of this strategy building process—key needs, capacity building blocks and main proposed activities—are presented in the following and should ideally be finalized by end of 2020, while all goals, related detailed tasks, implementation plan and quality control measures to operationalize the strategy during 2020–2030 will require further efforts to be presented in the final strategy document.

## Key needs and building blocks

The first step in building a European Exposure Science Strategy is the identification and assessment of exposure science key needs by considering major trends in Europe and elsewhere. To identify such key needs, two surveys were developed targeting exposure science professionals representing a wide spectrum of stakeholders. The first survey was conducted in 2016 and sent to all 560 participants attending the ISES 26^th^ Annual Meeting, 9–13 October, in Utrecht, The Netherlands. Participants were asked to provide their visions for exposure science in Europe in five and ten years, to identify the main drivers for exposure science, and how they see the role of a European chapter of ISES for improving exposure information and its policy uptake. First results and basic concepts were discussed among 142 participants during a dedicated workshop in support of setting up such a European ISES chapter.

The second survey was conducted in 2017 providing input from European exposure science practitioners in preparation of the first ISES Europe Workshop, held on 19–20 June 2018 in Dortmund, Germany. This workshop was hosted by the Federal Institute for Occupational Safety and Health (BAuA) and attended by 119 professionals from the European Commission, European agencies, national authorities, industry, academia, consultants, and insurance companies. In the preparatory survey, practitioners were asked for their top five priority areas for advancing exposure science in Europe until 2030. Reported data from both surveys were anonymised, pooled, and categorised using text analytics and expert judgement methods, which are discussed elsewhere (e.g. [58]). Based on analysing the survey results, six thematic focus areas for exposure science in Europe were identified, namely (1) data repositories and analytics, (2) regulatory exposure assessment, (3) exposure data production and monitoring, (4) building partnerships and collaboration, (5) exposure assessment methods and tools, and (6) exposure education and communication.

**Fig. 3:**
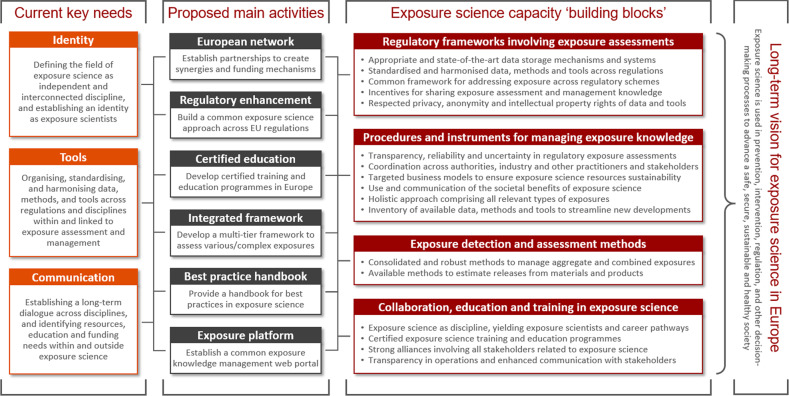
Key needs for exposure science in Europe, and capacity building blocks and related main proposed activities of a European Exposure Science Strategy to address these needs

These six thematic focus areas constitute the starting point for identifying key needs and main capacity building blocks for successfully developing a European Exposure Science Strategy. At the ISES Europe 2018 workshop, key needs and building blocks were derived by analysing strengths, weaknesses, opportunities, and threats of exposure science in Europe across the six focus areas [20]. Three overarching key needs have been identified, namely creating an exposure science ‘identity’, aligning exposure assessment and management ‘tools’, and advancing exposure science ‘communication’ (Fig. 3).

Creating an identity as an independent scientific discipline is crucial for defining and advancing the field’s focus areas, minimising overlaps and creating synergies. Such synergies should focus on connecting with adjacent disciplines, considering emerging research fields, and establishing networks and funding schemes. Aligning exposure assessment and management tools is required for a streamlined, efficient, and effective uptake of exposure science into policy, other relevant frameworks and strategies, and across various exposure science-related disciplines. Finally, advancing communication based on establishing a long-term dialogue among stakeholders from exposure science and adjacent disciplines will constitute the basis for effective knowledge transfer across stakeholders.

As an initial step to address the three identified key needs for exposure science in Europe, workshop participants discussed main capacity building blocks across the six thematic areas. Building blocks were identified along four domains, namely (a) regulations involving exposure assessment, material, facilities and activities, (b) exposure management tools, guidance, procedures and authorities, (c) assessment, detection, and monitoring of exposure, and (d) collaboration, education, training and communication of exposure science. Collaboration thereby should focus on European networks, but also involve international partners, such as the World Health Organization (WHO), the Organisation for Economic Co-operation and Development (OECD), and the United Nations Environment Programme (UN Environment), as well as countries that are strong in exposure science, such as the United States, Japan, Canada, and Australia. The specific building blocks are summarised in Fig. 3, each aimed at advancing, improving, and/or harmonising existing exposure science guidelines, regulations, practices, and information. Additional details are provided elsewhere [20]. The defined building blocks constitute the basis for specifying main strategic activities as foundation for a successful, broadly recognised and effective European Exposure Science Strategy.

## Main activities for a successful strategy

Exposure science has a promising future in Europe, involving a continuously growing and multi-disciplinary exposure science community. Current efforts focus on broadly agreeing on the identified needs and aligning exposure information across regulatory and non-regulatory frameworks and strategies. This requires a viable pool of scientists and practitioners that continuously develop and provide new and refine existing exposure information in line with the strategy objectives and priorities. Yet, creating a common identity as exposure scientists, establishing a cross-disciplinary and long-term dialogue between the various stakeholders, and the organisation and alignment of exposure science tools are the areas that urgently need to be addressed in order to create the necessary synergies and infrastructures. In response to these needs, six main areas of activities are proposed as long-term focus points for a successful strategy (see Fig. 3):

- *European network*. Establish European-wide partnerships in support of creating and maximising synergies and funding opportunities in exposure science. This will involve exposure scientists from different disciplines, policy-makers, practitioners and citizens, in order to exchange information relevant for the development, identification, promotion and exchange of best practices, education and training, and awareness-raising of the added value of exposure science.
- *Regulatory enhancement*. Build a common approach to exposure assessment and management with the aim to help enhance European policies and regulations that rely on exposure science. This will be achieved by addressing existing needs across policies and regulations, harmonising terms, vocabularies, and providing common templates for exposure information and documentation.
- *Certified education*. Develop tiered exposure science education and training programmes across Europe based on a cohesive set of learning objectives. Such programmes can eventually be certified and aligned with the European Credit Transfer and Accumulation System (ECTS), with ISES Europe as a facilitator for scientific development, educational training and instruction, and communication.
- *Integrated framework*. Develop a common and internally consistent framework for evaluating and selecting suitable exposure assessment data and models tailored towards distinct regulatory and non-regulatory questions requiring different tiers to assess various types (e.g. aggregate, cumulative, mixtures) and complexity levels of exposures across policy domains, based on harmonising and building consistency at various levels of refinement.
- *Best practices handbook*. Provide guidance for exposure scientists and practitioners by developing a handbook of exposure science and best practice, tailored to specific European needs and covering all relevant aspects including exposure scenarios, standardisation and harmonisation efforts, and a quality-rating system for data and models in use.
- *Exposure platform*. Establish a common web-based knowledge management portal for exchanging exposure science information in support of harmonising exposure assessment and management, and standardising data collection methods, tools and procedures.

These six key activities are strongly interlinked and complementary, and should therefore be all seen as essential elements in building a successful strategy. For example, information provided in the exposure platform in support of harmonising exposure assessment should be included in the best practice handbook to serve as guidance for practitioners, as well as in different tiers of exposure science education and training. All proposed activities are defined in support of Europe’s long-term goals and need to be translated into specific action plans. With that, these activities become feasible, given that related working groups can be established and be supported by European authorities and other organisations, which will provide a consistent way forward for exposure science in Europe.

## Conclusions and next steps

Establishing a European Exposure Science Strategy is an ongoing process that started with identifying key needs, published as outcome of the first ISES Europe workshop [20], specifying related building blocks and main proposed activity areas. In a next step, specific action plans will be defined along an aligned roadmap to address the identified key needs. Outcomes and a detailed implementation plan will be summarised in a Special Issue in the Journal of Exposure Science and Environmental Epidemiology. To facilitate the implementation of identified activities, a community of practice for European exposure science is currently being established through the formation of specific working groups. In this effort, ISES Europe will bring together stakeholders for exposure science in Europe, and as a society, the chapter will take the facilitator role for the working groups. Each of the working groups will further elaborate on developing its specific action plan and related tasks, and explore funding possibilities building on collaboration and knowledge exchange among various stakeholders. Proposals for new working group topics in line with the identified key needs are highly welcome and can be submitted to ISES Europe (see http://ises-europe.org/working-groups). Results from the working groups serve as critical element for a successful European Exposure Science Strategy with goals defined by 2020 and an iterative implementation of these goals and the action plan roadmap by 2020–2030, with specific objectives and concrete actions, and aligned with advances in science and technology.
